# Supplementary material for: Porphyromonas gingivalis fimbrial protein Mfa5 contains a von Willebrand factor domain and an intramolecular isopeptide
Source: Commun Biol. 2021 Jan 25;4:106. doi: 10.1038/s42003-020-01621-w (PMC7835359; doi:10.1038/s42003-020-01621-w)
Supplement: Supplementary file 2 — Supplementary Information [file 42003_2020_1621_MOESM2_ESM.pdf]

Supplementary information

Porphyromonas gingivalis fimbrial protein Mfa5 contains a von Willebrand factor domain and an intramolecular isopeptide

Thomas V. Heidler[1], Karin Ernits[1], Agnieszka Ziolkowska[1], Rolf Claesson[2] and Karina Persson[1*]

[1]Department of Chemistry, Umeå Centre for Microbial Research (UCMR), Umeå University, 90187, Umeå, Sweden.

[2]Department of Odontology, Umeå University, 90187 Umeå, Sweden.

* Corresponding author:

Tel: +46-90-7865926

E-mail: karina.persson@umu.se

This PDF includes

Supplementary Table 1

Supplementary Figures S1 to S6

Supplementary Table 1: Primer list.

Mfa5-1F	GAGAGAccatggGCATGATGAAACGATATACAATAATTC
Mfa5-21F	GAGAGAccatggGCTTTCAAATAAAAGCTCGCCCTTA
Mfa5-99F	TTTATTTTCAGGGCGccatgggcCGGGCATCTCCCCCCGTAGTT
Mfa5-138F	TTTATTTTCAGGGCGccatgGCGGTAACAGTACCTGTCGATGTGG
Mfa5-435R	GGTGGTGGTGctcgagTTAAGCTACAAAGTCCGTCACCTCCCC
Mfa5-664R	GGTGGTGGTGctcgagTTACCAATAATTGAGGAGCTTGTAAGCGAAGTAAG
Mfa5-1044R	GGTGGTGGTGctcgagTTAGTCGAATCCGAACGAAAGACCCCCA
Mfa5-1228R	CTCTCTctcgagTTAGTTGACTACAACTTTCCTTA
Mfa5-K111AF	GTCGACgccTATGCGGTACCGGTTGCCAATCCAATGG
Mfa5-K111AR	CGCATAggcGTCGACGGCAACGGGAACTACGGGG

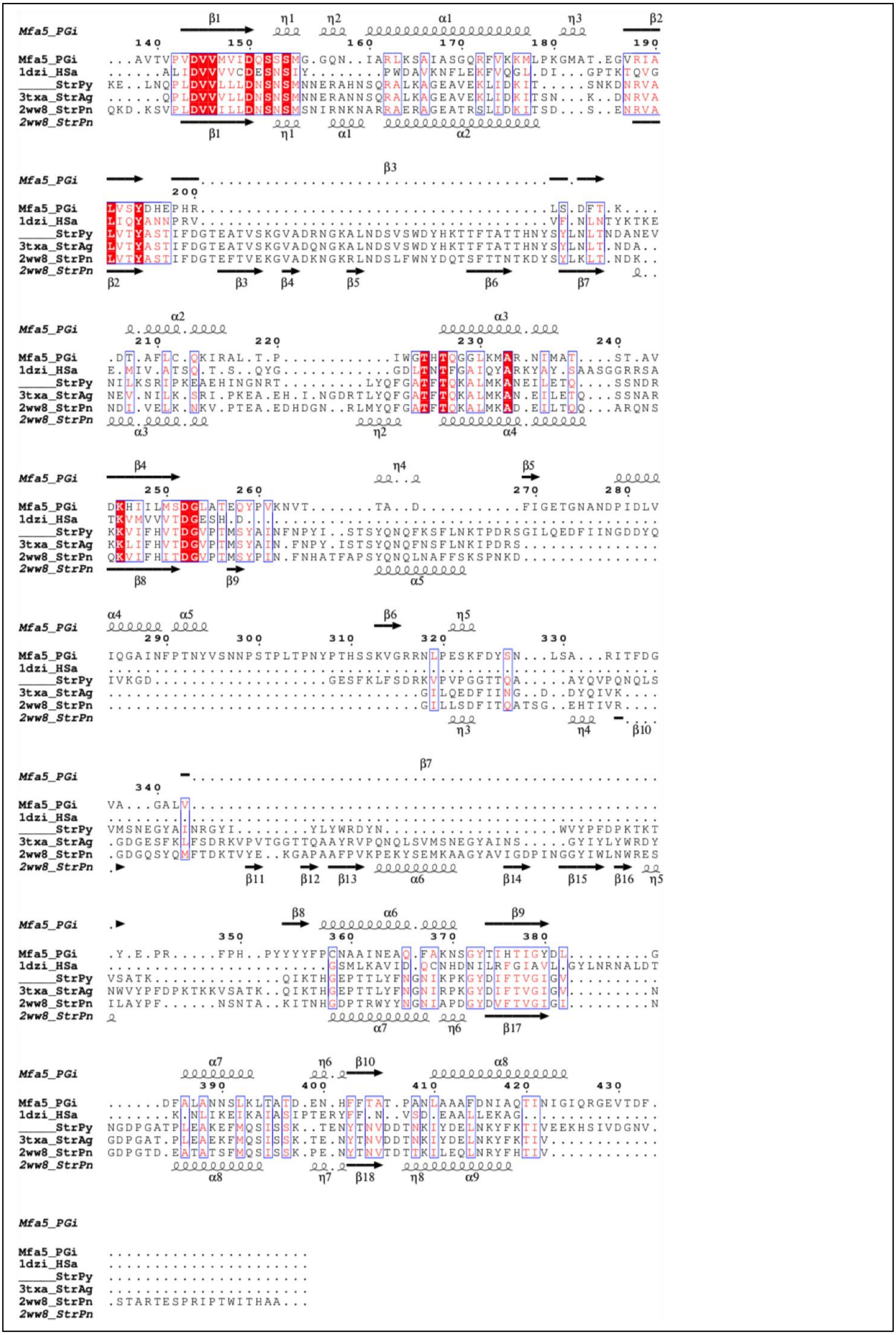
Mfa5_PGi
140 150 160 170 180 190
Mfa5_PGi ...AVTVPVDVVMVIDQSSSMG.GQN..IARLKSAIASGQRFVKKMLPKGMAT.EGVRIA
1dzi_HSa ......ALIDVVVVCDESNSIY........PWDAVKNFLEKFVQGL.DI...GPTKTQVG
_____StrPy KE..LNQPLDVVLLLDNSNSMNNERAHNSQRALKAGEAVEKLIDKIT.....SNKDNRVA
3txa_StrAg ......QPLDVVVLLDNSNSMNNERANNSQRALKAGEAVEKLIDKITSN...K..DNRVA
2ww8_StrPn QKD.KSVPLDVVILLDNSNSMSNIRNKNARRAERAGEATRSLIDKITSD...S..ENRVA
2ww8_StrPn
Mfa5_PGi
200
Mfa5_PGi LVSYDHEPHR..................................LS.DFT.K....
1dzi_HSa LIQYANNPRV..................................VF.NLNTYKTKE
_____StrPy LVTYASTIFDGTEATVSKGVADRNGKALNDSVSWDYHKTTFTATTHNYSYLNLTNDANEV
3txa_StrAg LVTYASTIFDGTEATVSKGVADQNGKALNDSVSWDYHKTTFTATTHNYSYLNLT.NDA..
2ww8_StrPn LVTYASTIFDGTEFTVEKGVADKNGKRLNDSLFWNYDQTSFTTNTKDYSYLKLT.NDK..
2ww8_StrPn
Mfa5_PGi
210 220 230 240
Mfa5_PGi .DT.AFLC.QKIRAL.T.P............IWGTHTQGGLKMAR.NIMAT....ST.AV
1dzi_HSa E.MIV.ATSQ.T.S..QYG...........GDLTNTFGAIQYARKYAY.SAASGGRRSA
_____StrPy NILKSRIPKEAEHINGNRT.........LYQFGATFTQKALMKANEILETQ....SSNDR
3txa_StrAg NEV.NILK.SRI.PKEA.EH.I.NGDRTLYQFGATFTQKALMKAN.EILETQ...SSNAR
2ww8_StrPn NDI.VELK.NKV.PTEA.EDHDGN..RLMYQFGATFTQKALMKAD.EILTQQ...ARQNS
2ww8_StrPn
Mfa5_PGi
250 260 270 280
Mfa5_PGi DKHIILMSDGLATEQYPVKNVT.......TA..D...........FIGETGNANDPIDLV
1dzi_HSa TKVMVVVTDGESH.D...
_____StrPy KKVIFHVTDGVPTMSYAINFNPYI..STSYQNQFKSFLNKTPDRSGILQEDFIINGDDYQ
3txa_StrAg KKLIFHVTDGVPTMSYAIN.FNPY.ISTSYQNQFNSFLNKIPDRS...............
2ww8_StrPn QKVIFHITDGVPTMSYPIN.FNHATFAPSYQNQLNAFFSKSPNKD...............
2ww8_StrPn
Mfa5_PGi
290 300 310 320 330
Mfa5_PGi IQGAINFPTNYVSNNPSTPLTPNYPTHSSKVGRRNLPESKFDYSN...LSA...RITFDG
1dzi_HSa ....
_____StrPy IVKGD.................GESFKLFSDRKVPVPGGTTQA.....AYQVPQNQLS
3txa_StrAg ...........................GILQEDFIING..D..DYQIVK....
2ww8_StrPn ...........................GILLSDFITQATSG..EHTIVR....
2ww8_StrPn
Mfa5_PGi
340
Mfa5_PGi VA...GALV...
1dzi_HSa ....
_____StrPy VMSNEGYAINRGYI.......YLYWRDYN.................WVYPFDPKTKT
3txa_StrAg .GDGESFKLFSDRKVPVTGGTTQAAYRVPQNQLSVMSNEGYAINS.....GYIYLYWRDY
2ww8_StrPn .GDGQSYQMFTDKTVYE..KGPAAFPVKPEKYSEMKAAGYAVIGDPINGGYIWLNWRES
2ww8_StrPn
Mfa5_PGi
350 360 370 380
Mfa5_PGi .Y.E.PR.....FPH..PYYYYFPCNAAINEAQ.FAKNSGYTIHTIGYDL.........G
1dzi_HSa ........................GSMLKAVID.QCNHDNILRFGIAVL.GYLNRNALDT
_____StrPy VSATK.............QIKTHGEPTTLYFNGNIKPKGYDIFTVGIGV..........
3txa_StrAg NWVYPFDPKTKKVSATK..QIKTHGEPTTLYFNGNIRPKGYDIFTVGIGV.........N
2ww8_StrPn ILAYPF......NSNTA..KITNHGDPTRWYYNGNIAPDGYDVFTVGIGI.........N
2ww8_StrPn
Mfa5_PGi
390 400 410 420 430
Mfa5_PGi ......DFALANNSLKLTATD.EN.HFFTAT.PANLAAAFDNIAQTINIGIQRGEVTDF.
1dzi_HSa .......K.NLIKEIKAIASIPTERYFF.N..VSD.EAALLEKAG..............
_____StrPy NGDPGATPLEAKEFMQSISSK..TENYTNVDDTNKIYDELNKYFKTIVEEKHSIVDGNV.
3txa_StrAg GDPGAT.PLEAEKFMQSISSK.TE.NYTNVDDTNKIYDELNKYFKTIV...........
2ww8_StrPn GDPGTD.EATATSFMQSISSK.PE.NYTNVTDTTKILEQLNRYFHTIV...........
2ww8_StrPn
Mfa5_PGi
Mfa5_PGi ....
1dzi_HSa ....
_____StrPy ....
3txa_StrAg ....
2ww8_StrPn .STARTESPRIPTWITHAA...
2ww8_StrPn

Supplementary Figure 1: Sequence alignment of the vWF domain. A structure-based sequence alignment of the Mfa5 vWF domain onto the streptococcal tip proteins RrgA (PDBID: 2ww8), GBS104 (PDBID: 3txa), and the integrin α2 I domain (PDBID: 1dzi) confirms the conserved MIDAS site (red) despite the overall low sequence similarity.

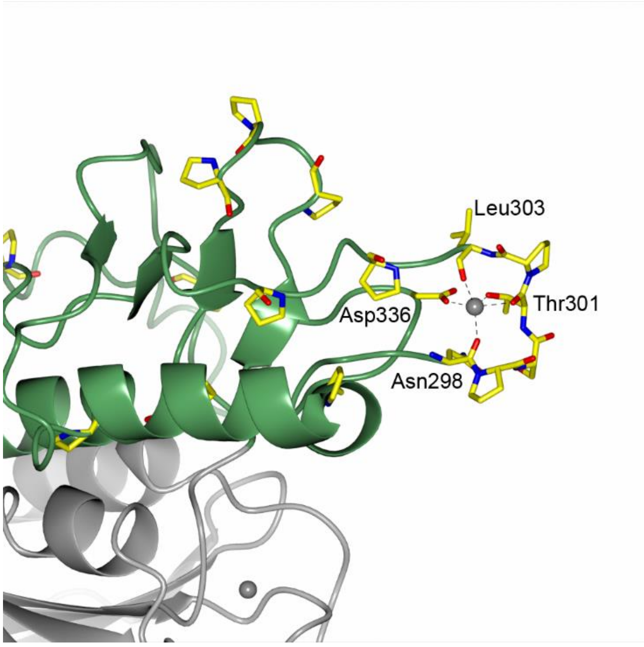

Supplementary Figure 2: The metal binding loop of the ARM2 domain. A metal (grey sphere) is coordinated by main chain and side chain oxygens. Coordinating residues and prolines are depicted as stick models. The interactions between the amino acid side chains and the metal are shown as dashed lines. The ARM2 domain is shown in green and the adjacent vWF domain in grey.

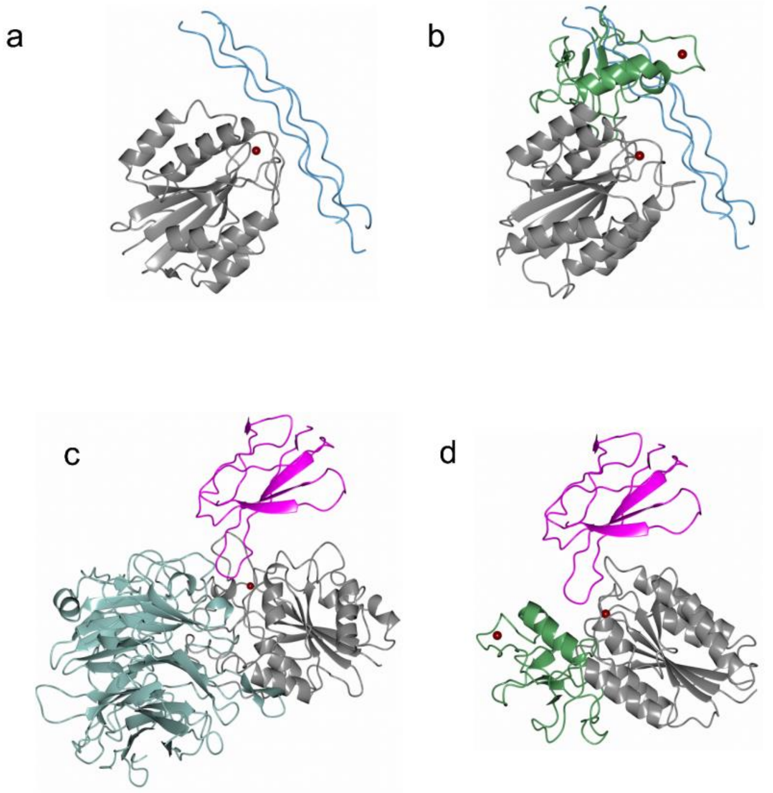

Supplementary Figure 3: Putative interaction with extracellular matrix molecules. a Collagen bound to human integrin α2-I. **b** Position of collagen when integrin α2-I was superimposed on the Mfa5 vWF domain. The ARM2 subdomain interferes with binding of the collagen triple helix. **c** Human integrin αVβ3 in complex with fibronectin. **d** Position of fibronectin and Mfa5 when integrin αVβ3 was superimposed on the Mfa5 vWF domain. The vWF domains in all structures are shown in grey, the Mfa5 ARM2 subdomain in green, the collagen triple helix in blue, the αV domain in sea green and fibronectin in magenta.

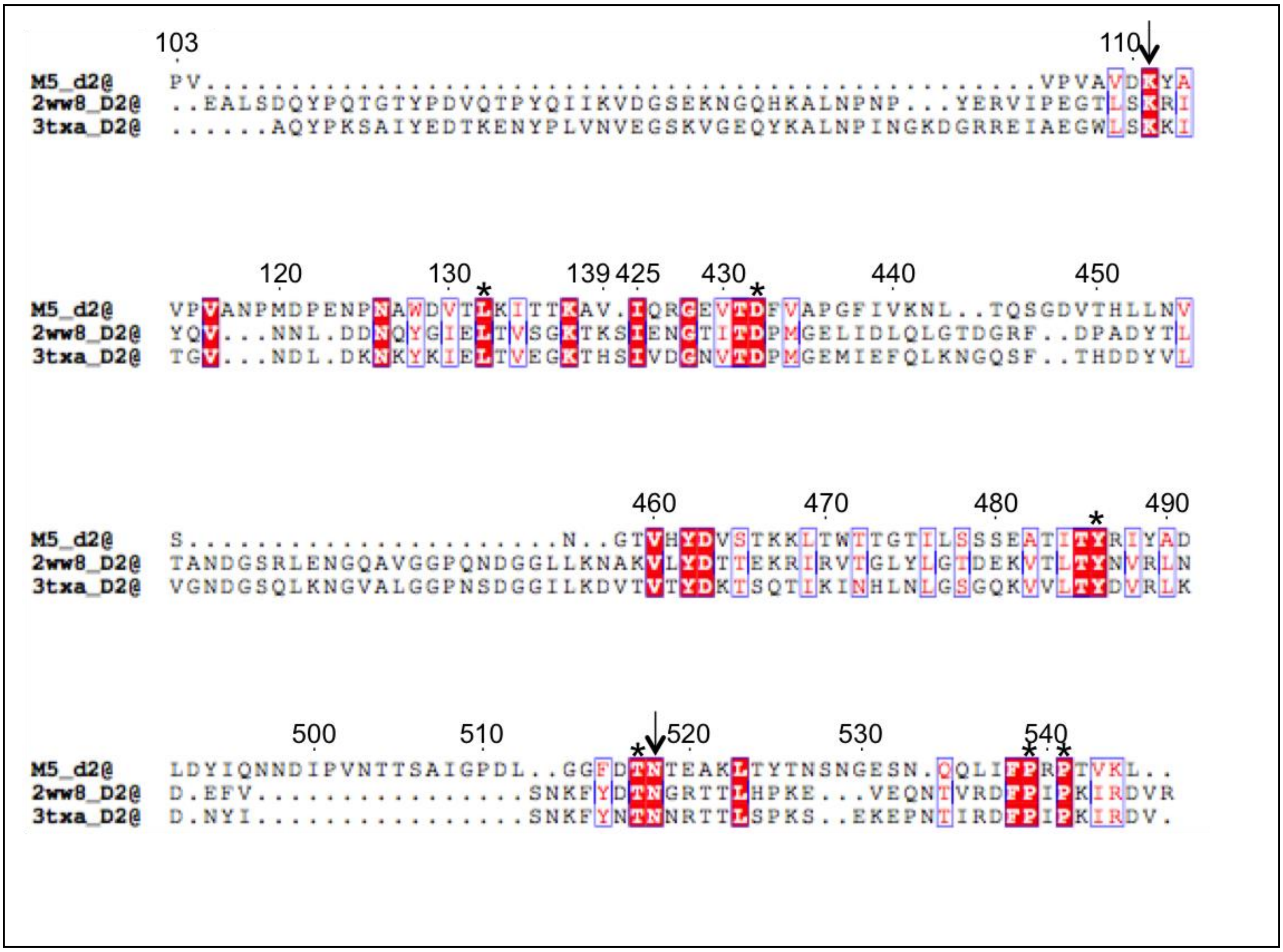

Supplementary Figure 4: Conserved isopeptide bond in domain 2. A structure-based sequence alignment of Mfa5 on RrgA and GBS104 shows the conserved residues forming the isopeptide bond (arrows) with its stabilizing residues (stars).

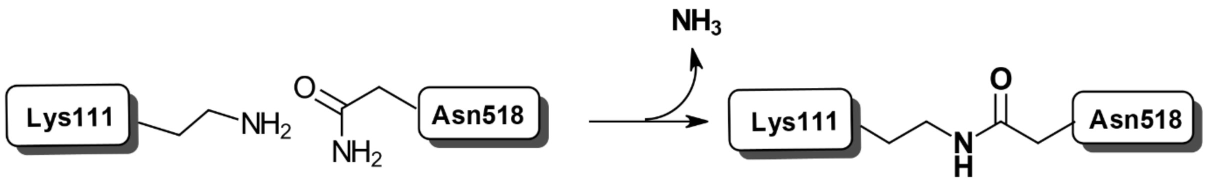

Supplementary Figure 5: Formation of an intramolecular isopeptide bond in Mfa5. The side chains of Lys111 and Asn518 react to form an amide bond. NH_3 is released in the reaction.

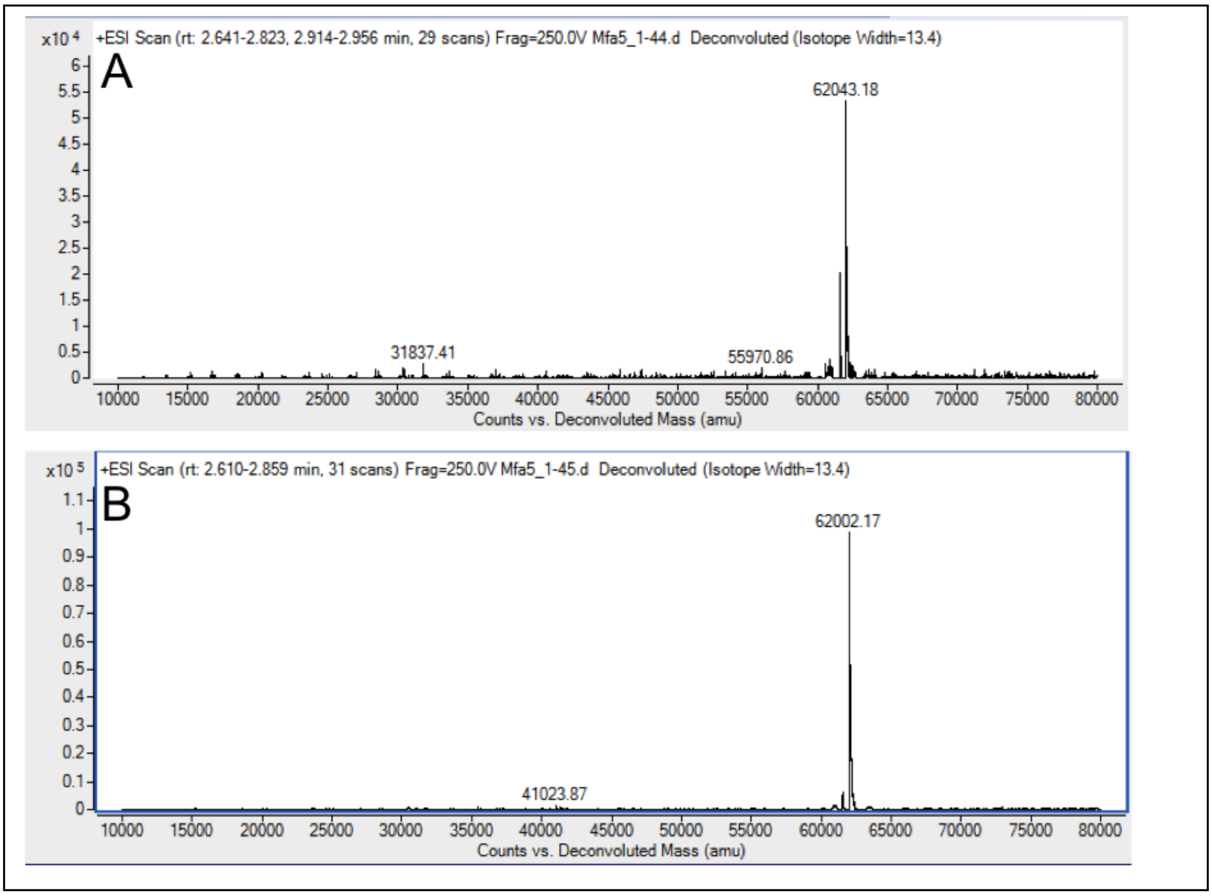

Supplementary Figure 6: ESI-TOF scan of wild type Mfa5 and its isopeptide mutant K111A. A) The wild type protein with the theoretical molecular mass of 62059.13 Da showed a mass of 62043 Da, and B) the K111A mutant showed a mass of 62002 Da, the exact theoretical value, indicating the loss of NH_3 upon formation of the isopeptide bond in the wild type.
