# Supplementary material for: Porphyromonas gingivalis fimbrial protein Mfa5 contains a von Willebrand factor domain and an intramolecular isopeptide
Source: Commun Biol. 2021 Jan 25;4:106. doi: 10.1038/s42003-020-01621-w (PMC7835359; doi:10.1038/s42003-020-01621-w)
Supplement: Supplementary file 3 — Description of Supplementary Files [file 42003_2020_1621_MOESM3_ESM.pdf]

Description of Additional Supplementary Files

File name: Supplementary Data 1

Description: Source data for Figure 5. Thermal shift assay data collected in triplicates. Data for Mfa5D1-D3 (K111) are shown as blue lines and Mfa5D1-D3 isopeptide mutant (K111A) as orange dotted lines. The minima represent the melting temperatures.
