# Supplementary material for: Porphyromonas gingivalis fimbrial protein Mfa5 contains a von Willebrand factor domain and an intramolecular isopeptide
Source: Commun Biol. 2021 Jan 25;4:106. doi: 10.1038/s42003-020-01621-w (PMC7835359; doi:10.1038/s42003-020-01621-w)
Supplement: Supplementary file 5 — Reporting Summary [file 42003_2020_1621_MOESM5_ESM.pdf]

nature research

Corresponding author(s): Karina Persson

Last updated by author(s): Jul 22, 2020

# Reporting Summary

Nature Research wishes to improve the reproducibility of the work that we publish. This form provides structure for consistency and transparency in reporting. For further information on Nature Research policies, see our Editorial Policies and the Editorial Policy Checklist.

## Statistics

For all statistical analyses, confirm that the following items are present in the figure legend, table legend, main text, or Methods section.

| n/a | Confirmed | |
|---|---|---|
| ☐ | ☒ | The exact sample size (*n*) for each experimental group/condition, given as a discrete number and unit of measurement |
| ☐ | ☒ | A statement on whether measurements were taken from distinct samples or whether the same sample was measured repeatedly |
| ☒ | ☐ | The statistical test(s) used AND whether they are one- or two-sided<br>*Only common tests should be described solely by name; describe more complex techniques in the Methods section.* |
| ☒ | ☐ | A description of all covariates tested |
| ☒ | ☐ | A description of any assumptions or corrections, such as tests of normality and adjustment for multiple comparisons |
| ☐ | ☒ | A full description of the statistical parameters including central tendency (e.g. means) or other basic estimates (e.g. regression coefficient) AND variation (e.g. standard deviation) or associated estimates of uncertainty (e.g. confidence intervals) |
| ☒ | ☐ | For null hypothesis testing, the test statistic (e.g. *F*, *t*, *r*) with confidence intervals, effect sizes, degrees of freedom and *P* value noted<br>*Give P values as exact values whenever suitable.* |
| ☒ | ☐ | For Bayesian analysis, information on the choice of priors and Markov chain Monte Carlo settings |
| ☒ | ☐ | For hierarchical and complex designs, identification of the appropriate level for tests and full reporting of outcomes |
| ☒ | ☐ | Estimates of effect sizes (e.g. Cohen's *d*, Pearson's *r*), indicating how they were calculated |

*Our web collection on statistics for biologists contains articles on many of the points above.*

## Software and code

Policy information about availability of computer code

| | |
|---|---|
| Data collection | The native dataset was collected at beamline MX14.1 at the Berlin Electron Storage Ring Society for Synchrotron Radiation (Germany). A high redundancy sulfur single-wavelength anomalous dispersion (SAD) dataset was collected at beamline ID29 at the European Synchrotron Radiation Facility (Grenoble, France) |
| Data analysis | All datasets were processed with XDS and the sulfur SAD data were combined with BLEND. Phase determination, refinement, and automated model building were performed in the PHENIX suite. The initial solution of Mfa5(138–435 )was used as a molecular replacement model for Mfa5(21–1044), which was built by BUCCANEER and COOT followed by refinement with PHENIX-refine. |

For manuscripts utilizing custom algorithms or software that are central to the research but not yet described in published literature, software must be made available to editors and reviewers. We strongly encourage code deposition in a community repository (e.g. GitHub). See the Nature Research guidelines for submitting code & software for further information.

## Data

Policy information about availability of data

All manuscripts must include a data availability statement. This statement should provide the following information, where applicable:

- Accession codes, unique identifiers, or web links for publicly available datasets
- A list of figures that have associated raw data
- A description of any restrictions on data availability

The protein structure coordinates and reflection data have been deposited in the protein data bank with the acession codes 6tnj and 6to1

# Field-specific reporting

Please select the one below that is the best fit for your research. If you are not sure, read the appropriate sections before making your selection.

☒ Life sciences ☐ Behavioural & social sciences ☐ Ecological, evolutionary & environmental sciences

For a reference copy of the document with all sections, see nature.com/documents/nr-reporting-summary-flat.pdf

# Life sciences study design

All studies must disclose on these points even when the disclosure is negative.

| | |
|---|---|
| Sample size | No sample size calculations were done. X-ray data were collected according to standard techniques. |
| Data exclusions | No data were excluded from the calculations of the X-ray structures. Standard crystallographic programs were used. |
| Replication | Crystals of the long version of Mfa5 in this paper can be reproduced. The crystals of the short version has not been reproduced. |
| Randomization | Randomization is not revelant for this study, which is a standard X-ray crystallography project. |
| Blinding | Blinding studies are not relevant in this study which relies on X-ray crystallographic data. |

# Reporting for specific materials, systems and methods

We require information from authors about some types of materials, experimental systems and methods used in many studies. Here, indicate whether each material, system or method listed is relevant to your study. If you are not sure if a list item applies to your research, read the appropriate section before selecting a response.

## Materials & experimental systems

| n/a | Involved in the study | |
|---|---|---|
| ☐ | ☒ | Antibodies |
| ☒ | ☐ | Eukaryotic cell lines |
| ☒ | ☐ | Palaeontology and archaeology |
| ☒ | ☐ | Animals and other organisms |
| ☒ | ☐ | Human research participants |
| ☒ | ☐ | Clinical data |
| ☒ | ☐ | Dual use research of concern |

## Methods

| n/a | Involved in the study | |
|---|---|---|
| ☒ | ☐ | ChIP-seq |
| ☒ | ☐ | Flow cytometry |
| ☒ | ☐ | MRI-based neuroimaging |

## Antibodies

| | |
|---|---|
| Antibodies used | Antibodies against Mfa5 was obtained from Agrisera, Sweden. The secondary antibody (donkey anti-rabbit) was obtained from abcam, Sweden #97061 |
| Validation | The Mfa5 antibody was validated using purified recombinant protein. The secondary antibody was validated by the company : https://www.abcam.com/donkey-rabbit-igg-hl-alkaline-phosphatase-ab97061.html |



April 2020
